# 951. The Value in Multidisciplinary Complex Outpatient Care Meetings

**DOI:** 10.1093/jbcr/irag033.518

**Published:** 2026-04-06

**Authors:** Bryan Bader

**Affiliations:** Regions Hospital, Saint Paul, MN

## Abstract

**Introduction:**

Our Burn Center meets weekly for multidisciplinary care meetings to discuss inpatients. However, these meetings do not continue once the patient discharges. We wanted to evaluate if adding multidisciplinary care meetings to discuss complex outpatients was valuable for both team members and the patient.

**Methods:**

Monthly, multidisciplinary team members within the burn center met to discuss 2-5 complex outpatients at each meeting. Multidisciplinary members include surgeons, advanced practice providers, nurses, occupational therapists, social workers, and psychotherapists.

Surveys measured team members' understanding of the patient's reconstructive/rehabilitation plan before and after the meetings; ^2^how useful the meeting was for the individual team member's role in the discussed patient's care; ^3^and if the team member felt the meeting improved coordination of care for the patient.

Interventions that were decided on in the meeting were also tracked and recorded. Interventions subtypes included psychosocial, therapy, coordination of care, and surgical.

**Results:**

All team members felt their understanding of the patient's reconstructive/rehabilitative plan remained the same or most often improved after the care meeting. The majority of team members felt the meeting was useful in their role in patient care. All team members felt the meeting improved the coordination of care for the patients discussed. All team members felt the meetings should continue as a regular part of outpatient burn care.

The majority of patients discussed had at least one immediate intervention made as a result of the meeting.

**Conclusions:**

Multidisciplinary complex outpatient care team meetings are beneficial to burn center team members and patients.

**Applicability of Research to Practice:**

Other burn centers should consider adding multidisciplinary meetings into their outpatient practice.

**Funding for the study:**

N/A.

